# A Silent Stroke in Disguise: Isolated Facial Numbness Preceding the Discovery of Multifocal Infarcts and Competing Etiologies

**DOI:** 10.7759/cureus.100529

**Published:** 2025-12-31

**Authors:** Bassem Al Hariri, Abdulqadir J Nashwan, Joudi Alhariri, Usamah Al-Anbagi, Osama Mohammed

**Affiliations:** 1 College of Medicine, Qatar University, Doha, QAT; 2 College of Medicine, Weill Cornell Medicine - Qatar, Doha, QAT; 3 Department of Internal Medicine, Hamad Medical Corporation, Doha, QAT; 4 Department of Nursing and Midwifery Research, Hamad Medical Corporation, Doha, QAT; 5 Department of Medical Education, Hamad Medical Corporation, Doha, QAT

**Keywords:** cardio embolism, carotid stenosis, etiology, incidental finding, ischemic stroke, neurocysticercosis, trigeminal sensory stroke

## Abstract

Effective secondary prevention depends on determining the exact cause of an ischemic stroke, but unusual presentations, such as localized facial numbness, might make the diagnosis difficult to make and confound the etiological research. We describe the case of a 47-year-old man who had a 12-day history of isolated perioral and tongue numbness and several untreated vascular risk factors. A neurological examination showed sensory deficiencies in the right hemi-tongue and the right V2 and V3 distributions of the trigeminal nerve. While magnetic resonance angiography (MRA) detected focal stenoses (>50%) in the left internal carotid artery, brain magnetic resonance imaging (MRI) showed acute multifocal infarcts in the right pons, right cerebral peduncle, left thalamus, and left occipitotemporal area. Transthoracic echocardiography was normal, but in the right occipital lobe, an incidental ring-enhancing lesion with a central "dot sign" was found, strongly suggesting neurocysticercosis (NCC). Because of his uncontrolled diabetes and the lesion's passive look, therapy for the incidental NCC was postponed while management focused on aggressively controlling his vascular risk factors and preventing additional strokes with antiplatelets and a statin. This case highlights the need for a hierarchical approach where management is guided by the most likely and modifiable thromboembolic sources rather than by asymptomatic incidental findings that could serve as "red herrings" and worsen underlying comorbidities.

## Introduction

Due to its various origins, which include cardioembolic stroke, large artery disease, and other uncommon causes, ischemic stroke in young and middle-aged adults frequently poses a diagnostic problem [1]. Atypical symptoms, such as isolated trigeminal sensory problems, are rare and can result in a delayed or incorrect diagnosis, whereas classic stroke diagnoses include motor or speech impairments [2]. About 2-5% of ischemic stroke presentations have trigeminal sensory complaints, which are most frequently linked to lacunar infarcts involving the brainstem or thalamus [2].

A known but uncommon cause of ischemic stroke is neurocysticercosis (NCC), a parasite infection brought on by the larval stage of Taenia solium that usually affects small perforating arteries through inflammatory vasculitis [3,4]. Although NCC is the most prevalent parasite infection of the central nervous system globally, especially in endemic areas, it is still an uncommon cause of stroke in non-endemic places [3]. It is uncommon for NCC to be accidentally found on stroke neuroimaging, which can make determining the cause more challenging [5].

To direct long-term secondary prevention, an accurate etiological diagnosis is crucial. The Trial of Org 10172 in Acute Stroke Treatment (TOAST) classification emphasizes the distinction between cardioembolic, small-vessel occlusion, and large-artery atherosclerosis [6]. A thorough, methodical workup is necessary when neuroimaging suggests several possible processes. We describe a complicated case with multifocal infarcts that manifested as an isolated sensory condition. The diagnosis and treatment of this patient were complicated by possible cardioembolic, symptomatic carotid stenosis and incidental NCC.

## Case presentation

A 47-year-old man presented to the outpatient clinic with a 12-day history of continuous, isolated numbness affecting his tongue and the perioral region. He specifically denied experiencing any weakness, visual abnormalities, speech difficulties, headaches, gait instability, or seizures, although he reported secondary discomfort from his dentures due to altered sensation. His medical history was significant for uncontrolled type 2 diabetes mellitus (last HbA1c 9.2%), hypertension (without medication), and hypertriglyceridemia. He claimed to have used alcohol in the past and to be an active smoker. He was not taking any medications at presentation.

Vital signs on admission were as follows: blood pressure was 158/96 mmHg, heart rate 78 bpm, and temperature 36.8°C. Random blood glucose was 14 mmol/L (252 mg/dL; normal fasting range: 3.9-5.6 mmol/L or 70-100 mg/dL). Complete blood count and basic metabolic panel were within normal limits. The lipid panel showed triglycerides of 4.8 mmol/L (normal: <1.7 mmol/L) and LDL cholesterol of 3.9 mmol/L (optimal: <2.6 mmol/L).

Neurological examination revealed significantly reduced pinprick and light touch sensation on the right side of the tongue and in the right V2 and V3 distributions of the trigeminal nerve. Two-point discrimination was impaired in the same regions. The remainder of the cranial nerve examination, motor strength, coordination, and gait were normal.

**Figure 1:**
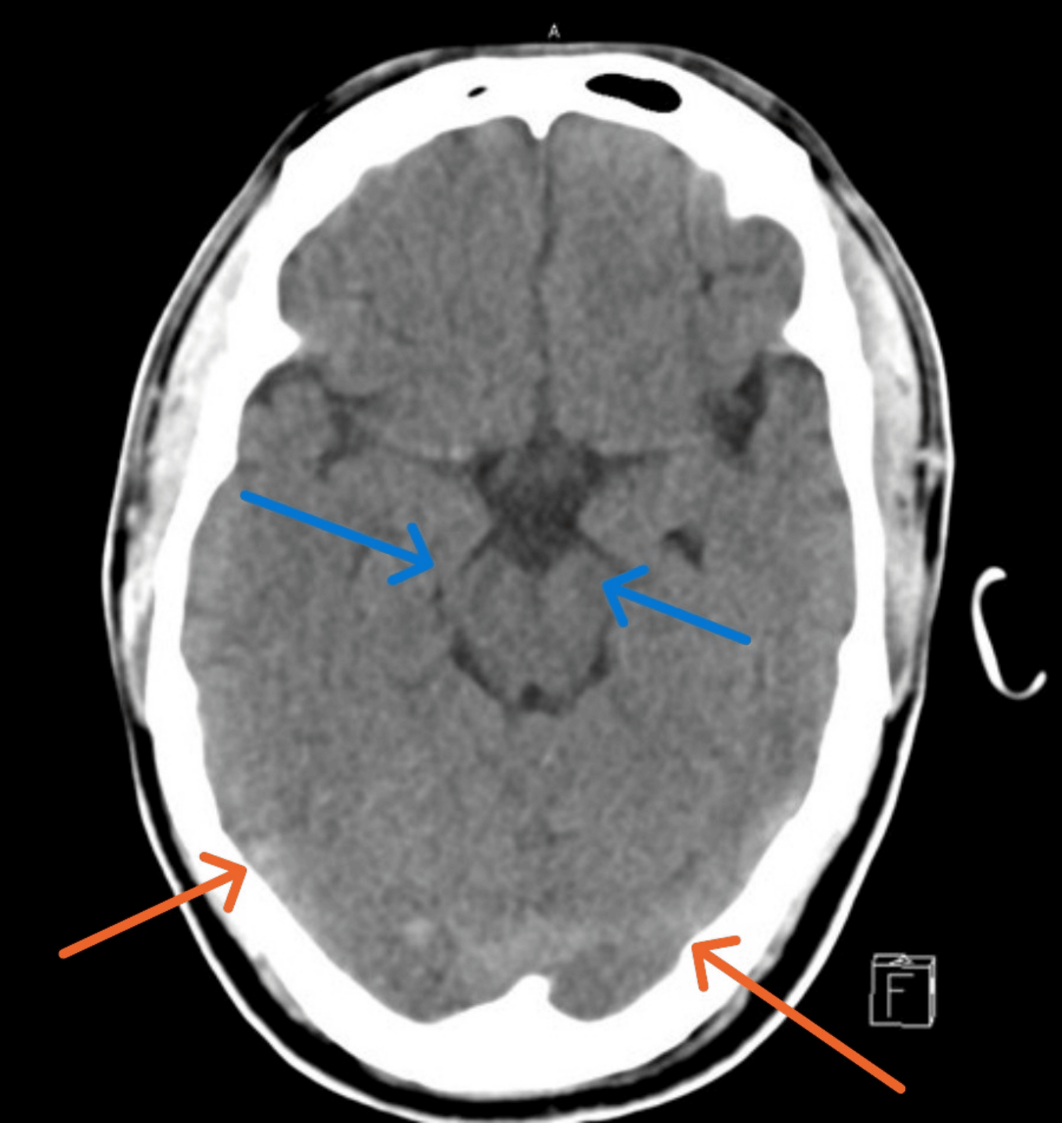
Head CT shows brain stem, thalamus, basal ganglia (blue arrows), and bilateral subcortical hypodensities suggesting ischemic insults (orange arrows).

CT head (non-contrast) revealed small hypodensities in the subcortical white matter, basal ganglia, left thalamus, and brainstem, suggestive of chronic ischemic changes (Figure 1).

**Figure 2:**
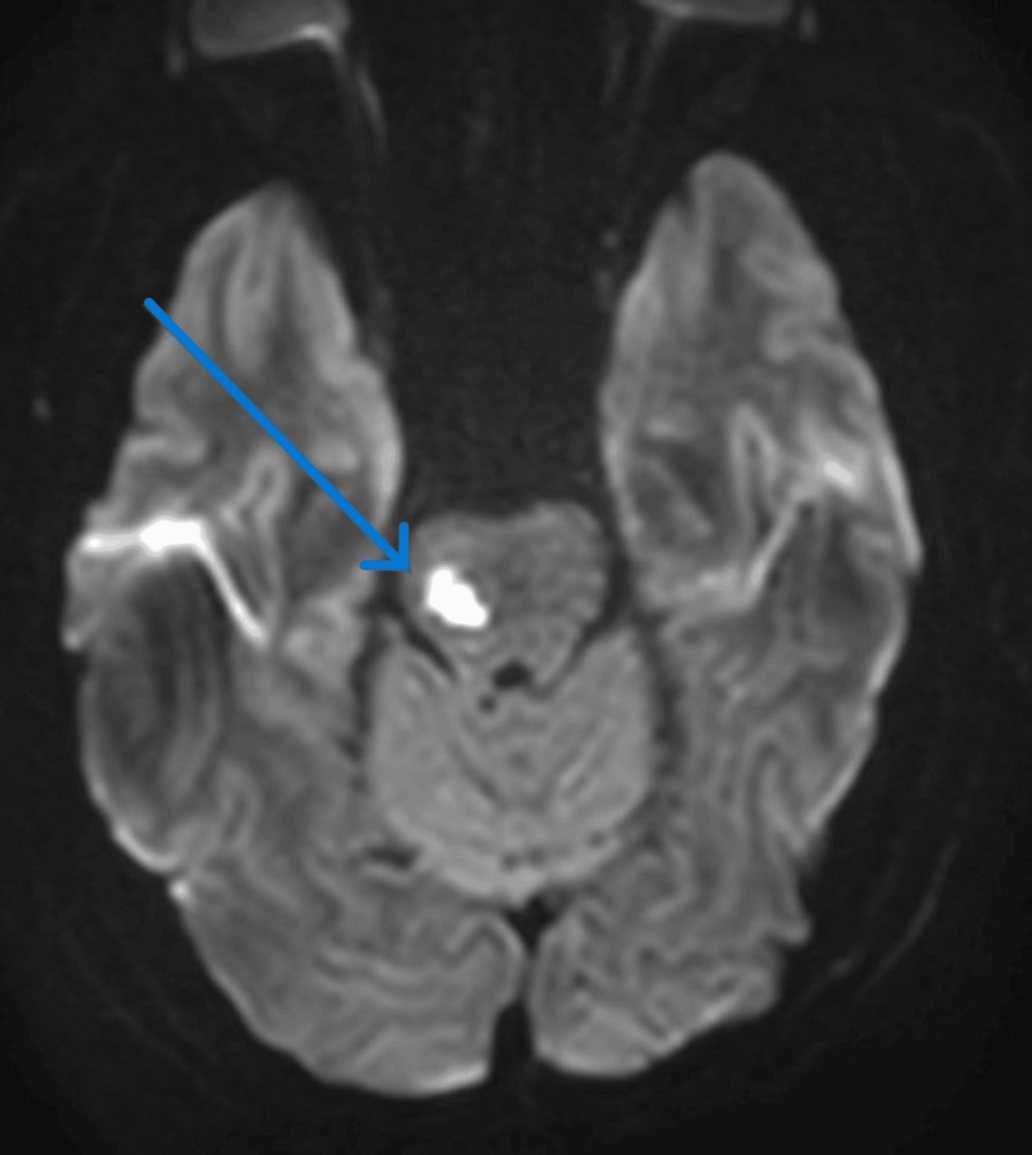
Head MRI shows multiple recent infarcts in the right midbrain (blue arrow).

**Figure 3:**
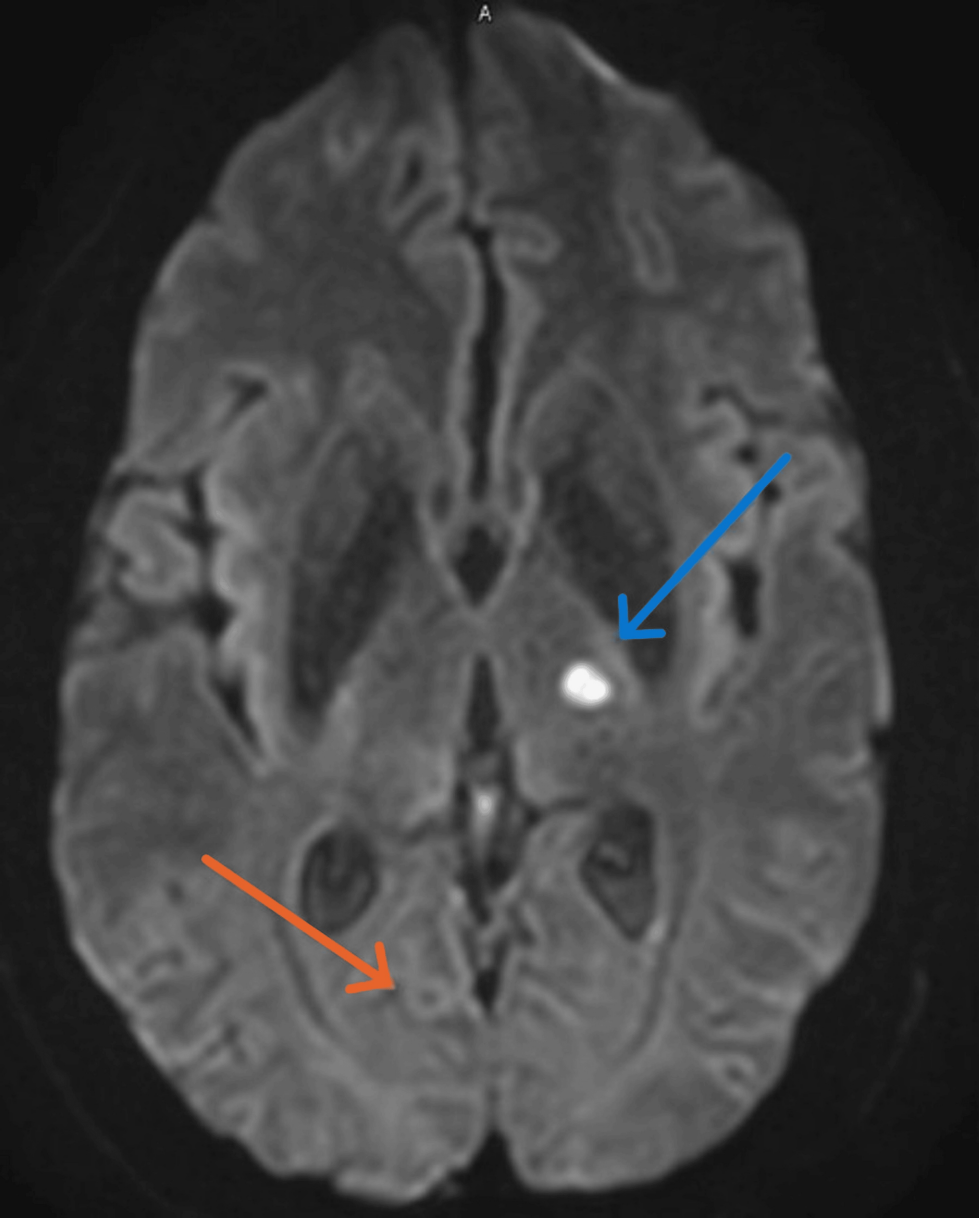
Head MRI shows left thalamus infracts (blue arrow), ring-enhancing lesion in the right occipital cortex exhibiting a central "dot sign," highly suggestive of neurocysticercosis (orange arrow).

MRI brain confirmed multiple acute infarcts, located in the right pons, right cerebral peduncle, left occipitotemporal white matter, and left thalamus (Figures 2, 3). Mild small vessel disease was also noted. A post-contrast sequence identified a 6 mm ring-enhancing lesion in the right occipital cortex exhibiting a central "dot sign," highly suggestive of neurocysticercosis (Figure 3). There was no significant perilesional edema.

**Figure 4:**
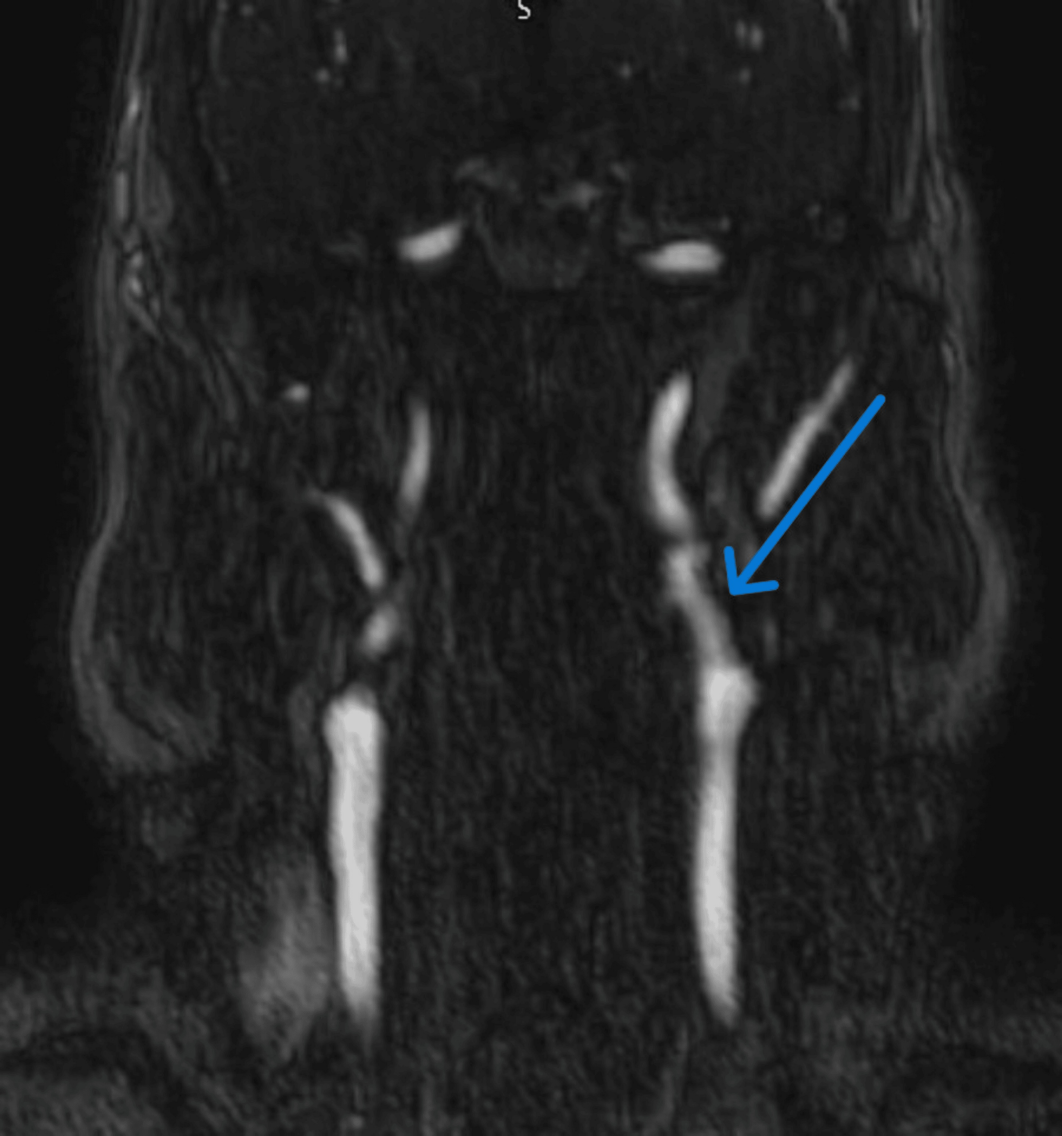
MRA Head & Neck shows focal stenosis (>50%) at the origin and cavernous segment of the left internal carotid artery (blue arrow).

MRA head & neck demonstrated focal stenosis exceeding 50% at the origin and cavernous segment of the left internal carotid artery (Figure 4).

**Figure 5:**
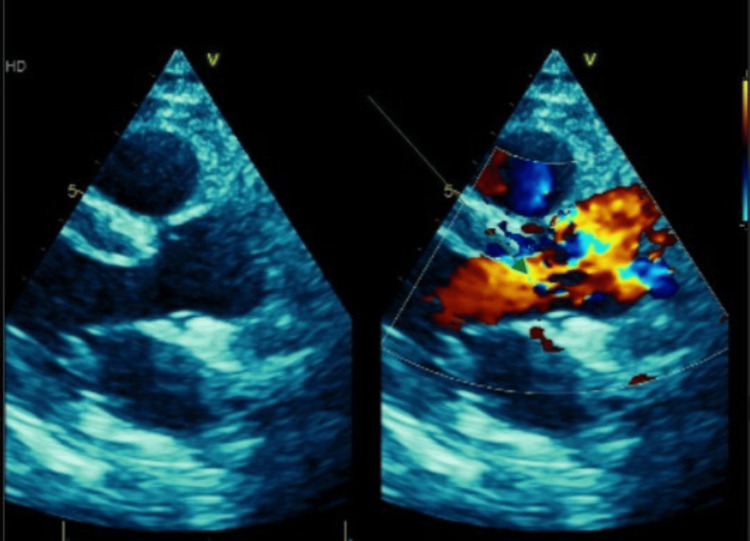
Transthoracic echocardiography showed normal systolic function, no intracardiac thrombus, and no patent foramen oval. Mild aortic regurgitation was observed (green arrow).

Transthoracic echocardiography showed normal systolic function (left ventricular ejection fraction 61%), no wall motion abnormalities, no intracardiac thrombus, and no patent foramen oval (Figure 5). Mild aortic regurgitation was observed.

The patient was diagnosed with an acute multifocal ischemic stroke. The widespread pattern of infarcts across both anterior and posterior circulations initially raised strong suspicion for a cardioembolic source. However, the clinical picture was further complicated by the coexistence of significant left internal carotid artery stenosis and an NCC lesion. A multidisciplinary team involving neurology, infectious disease, and cardiology was convened to formulate a hierarchical management plan.

Management was initiated as follows: The patient was started on dual antiplatelet therapy with aspirin (100 mg daily) and clopidogrel (75 mg daily), with a plan to de-escalate to aspirin monotherapy after three months, alongside high-intensity atorvastatin (40 mg daily). Glycemic control was optimized with subcutaneous insulin, and antihypertensive therapy was initiated with lisinopril 2.5 mg orally daily and amlodipine 5 mg orally daily. To further evaluate a possible cardioembolic etiology, a 30-day outpatient cardiac event monitor and a transesophageal echocardiogram were arranged.

The incidental NCC lesion prompted consultation with infectious diseases. Serological testing for cysticercosis (ELISA and immunoblot) was sent and later returned positive, supporting the imaging diagnosis. Treatment with antiparasitic therapy (albendazole) combined with high-dose corticosteroids was discussed but deliberately deferred given the lesion's appearance as a solitary, calcified, inactive cyst and the substantial risks posed by corticosteroid-induced hyperglycemia in a patient with poorly controlled diabetes.

Ophthalmology evaluation revealed no ocular cysts. Seizure prophylaxis was not initiated due to the absence of any seizure history. The patient was discharged after five days in a stable neurological condition. Coordinated follow-up was arranged across neurology, infectious diseases, endocrinology, and primary care to ensure continuity of care and ongoing etiological evaluation.

## Discussion

This case illustrates two intersecting diagnostic challenges: an ischemic stroke presenting with an unusual sensory manifestation and a clinical scenario complicated by multiple potential etiologies. The patient’s sole symptom, isolated numbness of the tongue and trigeminal (V2/V3) distribution, represents a rare and often misleading presentation of stroke. Such purely sensory deficits are typically associated with small lacunar infarcts involving the pons or thalamus [2,7]. In this case, the pontine infarct likely affected the principal sensory trigeminal nucleus or its descending tract. At the same time, the thalamic lesion involved the ventral posteromedial nucleus, which relays facial sensation. This bilateral disruption of the trigeminal sensory pathway explains the unilateral facial symptoms via a central disconnection mechanism. This pattern underscores that stroke may present in subtle, “silent” forms. It highlights the need to maintain a high index of suspicion even in the absence of motor, visual, or speech deficits.

The etiological complexity added a second layer of diagnostic difficulty. Three plausible mechanisms emerged from the clinical and imaging data, requiring a structured, hierarchical approach to interpretation. The multifocal distribution of infarcts across both anterior and posterior circulations strongly suggested a cardioembolic source, making this the leading hypothesis [8]. Importantly, initial transthoracic echocardiography does not exclude aortic arch atheroma or paroxysmal atrial fibrillation; therefore, transesophageal echocardiography and prolonged cardiac monitoring remain essential steps in the workup [9]. In our patient, a 30-day cardiac monitor was arranged to rule out paroxysmal atrial fibrillation.

Concurrently, severe stenosis of the left internal carotid artery represented a probable contributor through artery-to-artery embolism, particularly given the patient’s profile as a middle-aged man with poorly controlled vascular risk factors [10]. The presence of both anterior and posterior circulation infarcts, however, makes a singular carotid source less likely, reinforcing the possibility of a cardioembolic or multifocal thrombotic etiology. The incidental NCC lesion presented a third, but far less likely, possibility. Although NCC-related vasculitis can produce lacunar infarcts [3,4], the patient’s lesions were widespread, involving both small and large territories, and the NCC cyst was calcified and inactive, lacking the edema characteristic of active inflammatory disease [11]. Furthermore, serological confirmation of NCC did not alter its classification as an incidental finding, as the infarct topography was inconsistent with typical NCC-related vasculitis, which typically involves small perforating arteries near the cyst. 

Thus, while a minor vascular inflammatory effect could not be entirely excluded, NCC was considered incidental rather than causative. This diagnostic hierarchy directly shaped the management strategy. Deferring antiparasitic therapy for NCC was justified on clinical and safety grounds: initiating high-dose corticosteroids in a patient with uncontrolled diabetes carries a substantial risk of precipitating severe hyperglycemia or metabolic decompensation [12], while treating a solitary, calcified, asymptomatic lesion offers little proven benefit [11,13]. This approach aligns with the "primacy of the most modifiable risk" principle in stroke secondary prevention. This decision exemplifies the principle of primum non nocere, emphasizing the need to prioritize interventions that address the most immediate and modifiable threats while avoiding unnecessary treatment of incidental findings that may cause more harm than benefit [14].

## Conclusions

This case highlights that ischemic stroke can present with rare, isolated sensory symptoms, demanding clinical vigilance. In patients with multifocal strokes and multiple potential etiologies, a systematic, hierarchical diagnostic approach is essential. While uncommon conditions like NCC must be considered, therapy should first address the most probable and modifiable mechanism in this case, thromboembolism from a cardiac or carotid source, exacerbated by a profound metabolic syndrome. The decision to defer treatment for an incidental, inactive NCC lesion underscores the importance of patient-centered, risk-adjusted management. The management of incidental findings must be carefully integrated into the overall patient profile, ensuring that treatment for one condition does not inadvertently exacerbate another.

## References

[1] Béjot Y, Delpont B, Giroud M (2016). Rising stroke incidence in young adults: more epidemiological evidence, more questions to be answered. J Am Heart Assoc.

[2] Kim JS, Choi-Kwon S (1999). Sensory sequelae of medullary infarction: differences between lateral and medial medullary syndrome. Stroke.

[3] Cantú C, Barinagarrementeria F (1996). Cerebrovascular complications of neurocysticercosis. Clinical and neuroimaging spectrum. Arch Neurol.

[4] Barinagarrementeria F, Cantú C (1998). Frequency of cerebral arteritis in subarachnoid cysticercosis: an angiographic study. Stroke.

[5] Montano SM, Villaran MV, Ylquimiche L (2005). Neurocysticercosis: association between seizures, serology, and brain CT in rural Peru. Neurology.

[6] Adams HP Jr, Bendixen BH, Kappelle LJ, Biller J, Love BB, Gordon DL, Marsh EE 3rd (1993). Classification of subtype of acute ischemic stroke. Definitions for use in a multicenter clinical trial. TOAST. Trial of Org 10172 in Acute Stroke Treatment. Stroke.

[7] Bassetti C, Bogousslavsky J, Barth A, Regli F (1996). Isolated infarcts of the pons. Neurology.

[8] Kidwell CS, Wintermark M, De Silva DA (2013). Multiparametric MRI and CT models of infarct core and favorable penumbral imaging patterns in acute ischemic stroke. Stroke.

[9] Sanna T, Diener HC, Passman RS (2014). Cryptogenic stroke and underlying atrial fibrillation. N Engl J Med.

[10] (1991). North American Symptomatic Carotid Endarterectomy Trial. Methods, patient characteristics, and progress. Stroke.

[11] Nash TE, Garcia HH (2011). Diagnosis and treatment of neurocysticercosis. Nat Rev Neurol.

[12] Liu XX, Zhu XM, Miao Q, Ye HY, Zhang ZY, Li YM (2014). Hyperglycemia induced by glucocorticoids in nondiabetic patients: a meta-analysis. Ann Nutr Metab.

[13] Baird RA, Wiebe S, Zunt JR, Halperin JJ, Gronseth G, Roos KL (2013). Evidence-based guideline: treatment of parenchymal neurocysticercosis [RETIRED]: report of the Guideline Development Subcommittee of the American Academy of Neurology. Neurology.

[14] Morris Z, Whiteley WN, Longstreth WT Jr (2009). Incidental findings on brain magnetic resonance imaging: systematic review and meta-analysis. BMJ.

